# Familial coaggregation and shared genetic loading of mental disorders and cardiovascular diseases

**DOI:** 10.1017/S0033291725101724

**Published:** 2026-05-28

**Authors:** Pei-Chun Chen, Yi-Jiun Pan, Mei-Chen Lin, Mei-Hsin Su, Chun-Chieh Fan, Chi-Shin Wu, Shi-Heng Wang

**Affiliations:** 1 National Center for Geriatrics and Welfare Research, National Health Research Institutes, Zhunan, Taiwan; 2 Department of Microbiology and Immunology, School of Medicine, College of Medicine, China Medical University, Taichung, Taiwan; 3 Department of Public Health, College of Public Health, China Medical University, Taichung, Taiwan; 4 Department of Psychiatry, Virginia Institute for Psychiatric Behavioral Genetics, Virginia Commonwealth University, Richmond, VA, USA; 5 Center for Population Neuroscience and Genetics, Laureate Institute for Brain Research, Tulsa, OK, USA; 6 Department of Radiology, School of Medicine, University of California San Diego, La Jolla, CA, USA; 7 Department of Psychiatry, National Taiwan University Hospital, Yunlin Branch, Douliu, Taiwan; 8 Department of Medical Research, China Medical University Hospital, China Medical University, Taichung, Taiwan

**Keywords:** cardiovascular disease, familial coaggregation, mental disorders, polygenic risk scores

## Abstract

**Background:**

The high comorbidity between mental and cardiovascular diseases is widely recognized, but the underlying mechanisms are not yet well understood. We investigated the familial coaggregation and shared genetic loading between these diseases.

**Methods:**

Using claims data from Taiwan’s universal health insurance program, we identified cohorts of 4,513,509 individuals with parental information and 3,330,181 with full-sibling information born between 1970 and 1999, and followed up until 2020. Genotyping was available for 106,796 unrelated participants from the Taiwan Biobank. Multiple logistic regression models were used to estimate the association of parental history, full-sibling history, and polygenic risk scores (PRSs) for severe mental illnesses (schizophrenia, bipolar disorder, major depressive disorder [MDD]) with the risk of myocardial infarction, stroke, peripheral arterial disease (PAD), and heart failure.

**Results:**

Individuals with a parental history of schizophrenia, bipolar disorder, or MDD were more likely to have stroke and PAD. Coaggregation in full-siblings was observed for MDD with myocardial infarction and heart failure, bipolar disorders with stroke, and schizophrenia with PAD. The PAD risk increased with more relatives affected by MDD; the magnitude of association was larger when both parents were affected than when either parent was affected, and when there were two affected siblings than in those with only one. MDD PRS was positively associated with the risk of myocardial infarction and PAD.

**Conclusions:**

This study revealed familial coaggregation between mental and cardiovascular diseases, and shared polygenic liability of MDD with cardiovascular diseases. Our findings suggest the potential benefit of family-based integrated screening and preventive strategies.

## Introduction

Mental and cardiovascular disease are major causes of disability and mortality worldwide (GBD 2019 Mental Disorders Collaborators, 2022; GBD 2021 Forecasting Collaborators, 2024). Depression has been identified as a risk factor of cardiovascular disease (Visseren et al., 2021) and accounted for the largest proportion of disability-adjusted life years (37.3%) due to mental disorder in 2019 (GBD 2019 Mental Disorders Collaborators, 2022). Accumulating evidence also indicates that severe mental illnesses, such as schizophrenia and bipolar disorder, are associated with increased morbidity and mortality from cardiovascular disease (Polcwiartek et al., 2024).

Although the underlying mechanisms linking severe mental illness and cardiovascular disease are not yet well understood (Polcwiartek et al., 2024), biological, behavioral, psychological, and genetic mechanisms are proposed as plausible pathways (Goldfarb et al., 2022). There is increasing knowledge about the shared genetic and environmental factors between mental illness and cardiovascular disease (Hagenaars et al., 2020; Meijsen et al., 2024; Rødevand et al., 2021; Veeneman et al., 2024). However, familial coaggregation, which reflects the shared genetic and environmental loadings within families, between the two groups of disorders has been rarely addressed in the existing studies. A systematic analysis of pairs of major mental and cardiovascular diseases from the same, representative population is particularly lacking. Such information could inform strategies for early diagnosis and effective prevention of these comorbid conditions.

Furthermore, although shared genetic loading between psychiatric and cardiovascular diseases has been reported in European populations (Meijsen et al., 2024; Veeneman et al., 2024), whether their findings translate to other countries and populations remains uncertain. Notably, a recent study revealed that the relative contribution of environmental and heritable causes appeared to vary among different pairs of mental and cardiovascular diseases (Meijsen et al., 2024), highlighting the importance of studying each disease pair specifically.

In the present study, we conducted two analyses to investigate familial coaggregation and shared genetic etiology underlying the comorbidity between three mental disorders (schizophrenia, bipolar disorder, and major depressive disorder [MDD]) and four cardiovascular diseases (myocardial infarction, stroke, peripheral arterial disease (PAD), and heart failure). First, leveraging nationwide data of a universal health insurance program in Taiwan, we evaluated the coexistence of these diseases within individuals and their coaggregation within families, considering relatives with varying degrees of genetic similarity. Second, to explore shared polygenetic liability, we examined the association of polygenic risk scores (PRSs) for each mental disorder with each cardiovascular disease using data from a population-based biobank study in Taiwan.

## Methods

The study was approved by the Central Regional Research Ethics Committee of the China Medical University, Taichung, Taiwan (CRREC-108-30).

### Familial coaggregation study

#### Study design and population

The National Health Insurance (NHI) program is a compulsory single-payer health insurance system implemented in 1995, offering comprehensive healthcare coverage to over 99% of Taiwan’s residents (National Health Insurance Administration. National Health Insurance Annual Report 2022–2023 (bilingual). This cohort study used linked data from multiple NHI datasets, in which inpatient and ambulatory medical claims, demographics, and NHI registration information for each beneficiary were obtained using unique and immutable personal identifiers.

The Registry for Beneficiaries contains details about the relationships between insured individuals and their dependents, allowing for accurate inference of family relationships (Wang et al., 2022, 2024). In Taiwan, only spouses and blood relatives qualify as dependents of an insured person; relatives in law, including stepparent and stepchildren, are not eligible as dependents of an insured person. Using the information of the identifiers and unique/immutable personal identifier numbers of the biological parent, child, grandparent, grandchild, and spouse, family information was ascertained. To obtain the correct familial relationship, we ascertained parent–child with an age difference of at least 12 years. The pedigree inferred from covered-insurance relationship has been validated with high accuracy (99% for father–child relationship and 98% for mother–child relationship), compared to the Maternal and Child Health database containing information on the unique/immutable personal identifier of infants and both the biological parents (Wang et al., 2022, 2024). We identified 4,513,509 individuals born between 1970 and 1999 with known biological parent–child relationships. Among these individuals, 3,330,181 were eligible for analysis of full siblings, defined as individuals who had the same paternal and maternal identifiers. The sample size for half-siblings is too limited (~30,000 individuals with paternal half-siblings and ~ 20,000 individuals with maternal half-siblings) to produce sufficiently precise estimates. Therefore, the familial coaggregation analyses in half-siblings were not considered in this study.

#### Ascertainment of mental and cardiovascular diseases

Using claims data for inpatient and outpatient care from 1998 to 2020, we identified study subjects ever diagnosed with mental and cardiovascular diseases, i.e., schizophrenia, bipolar disorder, MDD, myocardial infarction, stroke, PAD, and heart failure. The diseases and disorders recorded in the claims data were coded according to International Classification of Disease (ICD); ICD-9 codes were used from 1998 to 2015, and ICD-10 codes from 2016 to 2020 (see Supplementary Table S1).

#### Statistical analysis

The number and percentage of study subjects for categorical demographic variables, and the prevalence of mental and cardiovascular diseases, were reported for both the entire cohort and cohort with full-sibling data. In all within-individual and familial coaggregation analyses, we used generalized estimating equations with an exchangeable working correlation structure to account for the nonindependence of data within the family clusters. All analyses were conducted separately for each pair of mental and cardiovascular diseases. Odds ratios (ORs) and 95% confidence intervals (CIs) were estimated using the generalized linear model with a binomial distribution and logistic link function. The within-individual comorbidity analysis provided the ORs (95% CIs) for cardiovascular diseases comparing study subjects with and without mental disorders, adjusting for sex, birth cohort, age, income level (monthly insured salary), and urbanization level.

Two familial coaggregation analyses were performed to evaluate whether individuals with relatives diagnosed with mental disorders are more likely to have cardiovascular diseases. First, the ORs (95% CI) for cardiovascular diseases were estimated for individuals with parental histories of mental disorders (affected father only, affected mother only, both parents) compared with those with unaffected parents, adjusting for sex, birth cohort, age, income level, urbanization level, father’s age, mother’s age, and the individual’s mental disorders. Second, we assessed whether individuals with full siblings diagnosed with mental disorders have increased odds of cardiovascular diseases compared to those with unaffected siblings. To evaluate the extent of sibling coaggregation, we categorized the number of affected siblings into 0 (reference group), 1, and ≥ 2, and repeated the regression analyses, adjusting for sex, birth cohort, age, income level, urbanization level, sibling’s age, sibling size, and the individual’s mental disorders. All statistical analysis were performed using SAS 9.4 (SAS Institute Inc., Cary, NC, USA). A P value less than 0.05 was considered statistically significant.

### Polygenic risk scores (PRSs)-association study

#### Study design and population

The Taiwan Biobank study (TWB) is a prospective community-based study that recruits participants aged 30–70 years without history of cancer across Taiwan (Chen et al., 2016; Fan, Lin, & Lee, 2008; Wu et al., 2024). Each participant signed an informed consent form, participated in face-to-face interviews, and provided blood samples. The TWB was linked to NHI claims datasets to collect information on diseases and disorders. The ascertainment of mental and cardiovascular diseases followed the same methodology as described in the familial coaggregation study.

#### Genotyping, quality control, and PRS calculation

From TWB, we obtained genome-wide genotyping for 131,048 study subjects. Genome-wide genotyping was performed using custom Taiwan Biobank chips run on the Axiom Genome-Wide Array Plate System (Affymetrix, Santa Clara, CA, USA). Variants with a call rate < 5%, minor allele frequency < 0.001, and deviation from Hardy–Weinberg equilibrium with *p* < 1E-06 were excluded. We used the 504 EAS panel from the 1000 Genomes Project and the 973 TWB panel from whole-genome sequencing in TWB participants as the reference panel to impute the genotypes with IMPUTE2; variants with imputation info >0.7 were retained.

After excluding duplicated samples, non-EAS samples, samples with a missing rate of more than 2%, or heterozygosity outliers (exceeding 5 standard deviation), 128,775 samples remained. To remove cryptic relatedness, one of the study participants was removed if pair-wise participants with PI-HAT >0.1875; 106,806 unrelated participants were kept and linked to NHI claims datasets, resulting in a cohort of 106,796 participants available for data analyses. For each mental disorder PRS analysis, we excluded participants who had the corresponding mental disorders at baseline.

PRS for schizophrenia, bipolar disorder, and MDD were derived using PRS-CSx (if large-scale genome-wide association study (GWAS) summary was available in both European and East Asian populations) (Ruan et al., 2022), or PRS-CS (Ge et al., 2019). Specifically, we used data from the Psychiatric Genomics Consortium meta-analysis as a discovery sample to identify risk variants for schizophrenia, bipolar disorder, and MDD and calculated the PRS for each of the three mental disorders in TWB participants. To enhance the performance of PRS predictions, we utilized PRS-CSx, a cutting-edge Bayesian polygenic modeling method, to derive PRS for schizophrenia (Ruan et al., 2022). This method integrates GWAS summary statistics from different discovery samples, specifically GWAS data from 22,778 cases and 35,362 controls of East Asian descent (Lam et al., 2019) and GWAS data from 53,386 cases and 77,258 controls of European descent (Trubetskoy et al., 2022). This approach resulted in better predictive performance than using PRS derived solely from a predominantly European GWAS sample of 67,390 cases and 94,015 controls (Trubetskoy et al., 2022) in our target population. Similarly, for MDD, PRS-CSx was used to combine GWAS data from 13,042 cases and 88,467 controls of East Asian descent (Giannakopoulou et al., 2021) with GWAS data from 246,363 cases and 561,190 controls of European descent (Howard et al., 2019), achieving superior prediction compared to using only the European GWAS data. For bipolar disorder, PRS was derived from a GWAS sample of 41,917 cases and 371,549 controls of European descent using PRS-CS (Ge et al., 2019; Mullins et al., 2021), a polygenic prediction method that estimates posterior effect sizes of susceptibility variants through a high-dimensional Bayesian regression framework with continuous shrinkage priors.

#### Statistical analysis

Logistic regression models were used to calculate the ORs (95% CIs) for cardiovascular diseases associated with the PRS for schizophrenia, bipolar disorder, and MDD. Analyses were conducted separately for each pair of mental and cardiovascular diseases and adjusted for sex, age, batch version, and 20 population stratification dimensions. A *P* value less than 0.05 was considered statistically significant. For the significant PRS association, mediation analyses and stratified analyses were further performed. Blood pressure and metabolic traits have been recognized as modifiable factors that underlie the comorbidity between mental disorders and cardiovascular diseases (Bergstedt et al., 2024); the potential mediating role of blood pressure (including systolic and diastolic) and metabolic traits (including body mass index, high-density lipoprotein, low-density lipoprotein, total cholesterol, and triglycerides) in the PRS association was further evaluated by estimating the direct effect, indirect effect, and mediation proportion using the CAUSALMED procedure in SAS. The 95% CIs were estimated using bootstrap procedures. Individuals with low-density lipoprotein ≤1.4 mmol/L have been considered as very-high-risk group; hence, the low-density lipoprotein categories-stratified analyses were performed to evaluate whether achieving ultra-low low-density lipoprotein modifies the PRS effect.

## Results

### Familial coaggregation study

The demographic characteristics of the study subjects are shown in Table 1. Approximately, half of the total cohort were men (52.92%) and were born between 1990 and 1999 (45.74%). The prevalence of schizophrenia, bipolar disorder, and MDD in the total cohort was 0.85%, 1.09%, and 6.05%, respectively. The demographics and prevalence of mental disorders in the full-sibling cohort were similar to those in the total cohort.

**Table 1.** Distribution of the demographics, mental disorders, and cardiovascular diseases in the total cohort and full-sibling cohort identified from National Health Insurance claims data, Taiwan Table 1. long description.

Variables	Total cohort		Full-sibling	
	*N* = 4,513,509		*N* = 3,330,181	
	*n*	%	*n*	%
Sex				
Female	2125041	47.08	1608636	48.30
Male	2388468	52.92	1721545	51.70
Birth Cohort				
1970–1979	732669	16.23	391344	11.75
1980–1989	1716252	38.02	1378775	41.40
1990–1999	2064588	45.74	1560062	46.85
Income level, NTD				
<26400	903679	20.05	644710	19.38
26400–45800	1838065	40.78	1370104	41.19
>45800	1765498	39.17	1311104	39.42
Urbanization				
Urban	2462452	54.63	1776746	53.41
Sub-urban	1677108	37.21	1265802	38.05
Rural	368078	8.17	284308	8.55
Mental disorders				
Schizophrenia	38474	0.85	41186	1.24
Bipolar disorder	49351	1.09	52451	1.58
Major depressive disorder	272903	6.05	279726	8.40

*Note*: NTD, new Taiwan dollar.

Greater odds of coexisting cardiovascular diseases were observed among individuals with mental disorders for all disease pairs, except for schizophrenia and myocardial infarction, in the adjusted models (Table 2). In general, the associations of mental disorders with stroke and PAD were stronger than their associations with myocardial infarction and heart failure.

**Table 2.** Odds ratios of cardiovascular diseases in association with mental disorders (sample size = 4,513,509) Table 2. long description.

		No. of subjects	Myocardial infarction					Stroke					Peripheral arterial disease					Heart failure				
			*n*	%	aOR	LCL	UCL	*n*	%	aOR	LCL	UCL	*n*	%	aOR	LCL	UCL	*n*	%	aOR	LCL	UCL
SCZ	No	4475035	4388	0.10	1.00	–	–	16893	0.38	1.00	–	–	17671	0.39	1.00	–	–	8473	0.19	1.00	–	–
	Yes	38474	69	0.18	0.90	0.70	1.14	429	1.12	1.87	1.69	2.06	450	1.17	2.04	1.86	2.25	196	0.51	1.75	1.51	2.02
BPD	No	4464158	4373	0.10	1.00	–	–	16798	0.38	1.00	–	–	17567	0.39	1.00	–	–	8484	0.19	1.00	–	–
	Yes	49351	84	0.17	1.36	1.09	1.68	524	1.06	2.38	2.18	2.60	554	1.12	2.39	2.20	2.60	185	0.37	1.72	1.49	1.99
MDD	No	4240606	4047	0.10	1.00	–	–	14937	0.35	1.00	–	–	15452	0.36	1.00	–	–	7812	0.18	1.00	–	–
	Yes	272903	410	0.15	1.28	1.16	1.42	2385	0.87	2.17	2.07	2.26	2669	0.98	2.22	2.13	2.32	857	0.31	1.56	1.45	1.67

*Note*: aOR, adjusted odds ratio; BPD, bipolar disorder; LCL, lower confidence limit; MDD, major depressive disorder; SCZ, schizophrenia; UCL, upper confidence limit. The models were adjusted for sex, birth cohort, age, income level, and urbanization level.

**Figure 1.**
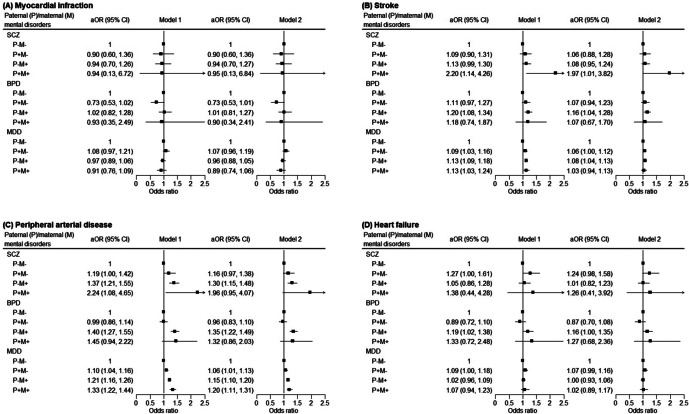
Odds ratios of cardiovascular diseases in association with parental history of mental disorders (sample size = 4,513,509). Model 1 was adjusted for sex, birth cohort, age, income level, urbanization level, father’s age, and mother’s age; model 2 was further adjusted for the individual’s own psychiatric diagnosis. *Note*: aOR, adjusted odds ratio; BPD, bipolar disorder; CI, confidence interval; MDD, major depressive disorder; SCZ, schizophrenia. Figure 1. long description.

The ORs for the associations between the parental history of mental disorders and the likelihood of having cardiovascular diseases are graphically displayed in Figure 1 (See Supplementary Tables 2–5 for details on the prevalence of cardiovascular diseases). Model 1 was adjusted for demographics and parental age; and model 2 was additionally adjusted for individual’s corresponding psychiatric diagnosis. Parental history of mental disorders was associated with increased odds of stroke and PAD (Figure 1b,c), but not with myocardial infarction or heart failure (Figure 1a,d). Generally, adjusting for individual’s psychiatric diagnosis led to a mild attenuation of the association, except for MDD and stroke, where the associations were markedly attenuated in Model 2. In models adjusted for all covariates, the OR for PAD was highest when both parents had MDD (1.20, 95% CI 1.11–1.31), followed by maternal MDD alone (1.15, 95% CI 1.10–1.20), and paternal MDD alone (1.06, 95% CI 1.01–1.13), compared with neither parents having MDD (Model 2, Figure 1c). Similar patterns were observed for a parental history of schizophrenia. Increased odds of stroke were observed in individuals with a parental history of schizophrenia in both parents, and in those with maternal histories of bipolar disorders or MDD (Model 2, Figure 1b).

**Figure 2.**
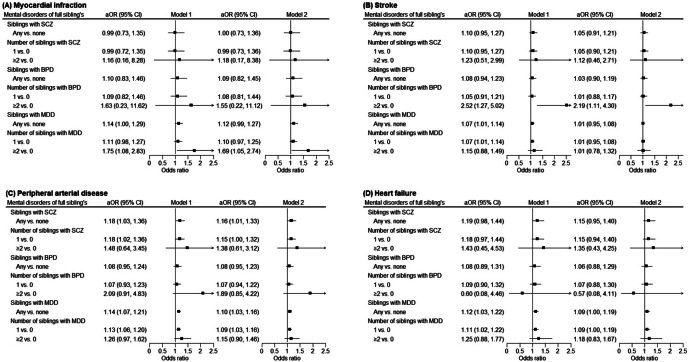
Odds ratios of cardiovascular diseases in association with full siblings’ history of mental disorders (sample size = 3,330,181). Model 1 was adjusted for sex, birth cohort, age, income level, urbanization level, sibling’s age, and sibling size; model 2 was further adjusted for the individual’s own psychiatric diagnosis. *Note*: aOR, adjusted odds ratio; BPD, bipolar disorder; CI, confidence interval; MDD, major depressive disorder; SCZ, schizophrenia. Figure 2. long description.

Figure 2 illustrates the adjusted ORs for cardiovascular diseases associated with a history of mental disorders in full siblings (See Supplementary Tables 6–9 for details on the prevalence of cardiovascular diseases). Having siblings with MDD was associated with increased odds of myocardial infarction (OR, 1.12; 95% CI, 0.99–1.27), PAD (OR, 1.10; 95% CI, 1.03–1.16) and heart failure (OR, 1.09; 95% CI, 1.00–1.19), and the ORs increased with number of affected siblings (Figure 2a,b,d; Model 2). An increased risk of stroke coaggregation with bipolar disorders was observed in individuals with multiple siblings affected by bipolar disorders, compared to those with no affected siblings (OR for stroke, 2.19; 95% CI, 1.11–4.30; Model 2, Figure 2b). Having siblings with schizophrenia was associated with increased risk of PAD (OR, 1.16; 95% CI, 1.01–1.33).

### Polygenic risk scores (PRSs)-association study

Descriptive statistics regarding age, sex, metabolic traits, and the prevalence of mental and cardiovascular disorders in 106,796 TWB participants are presented in Supplementary Table 10. We identified 106,127 participants for the PRS analysis of schizophrenia, 105,071 for bipolar disorder, and 92,828 for MDD after excluding those with the corresponding mental disorders at baseline.

The MDD PRS was associated with an increased risk of myocardial infarction and PAD after adjusting for sex, age, batch version, and 20 population stratification dimensions (OR [95% CI] per standard deviation increase in PRS: 1.09 [1.01–1.19] and 1.04 [1.00–1.08], respectively; Table 3). There was a borderline significant positive association between MDD PRS and stroke (1.04; 95% CI, 0.99–1.09; Table 3). In contrast, there was no evidence that schizophrenia PRS or bipolar disorder PRS was associated with any cardiovascular diseases. No clear evidence was observed for the associations between other mental and cardiovascular disease pairs.

**Table 3.** Odds ratios of cardiovascular diseases associated with per SD increase in polygenic risk score for mental disorders Table 3. long description.

	Myocardial infarction			Stroke			Peripheral arterial disease			Heart failure		
	aOR	LCL	UCL	aOR	LCL	UCL	aOR	LCL	UCL	aOR	LCL	UCL
SCZ PRS	1.00	0.93	1.08	1.01	0.96	1.05	0.99	0.96	1.02	0.94	0.87	1.02
BPD PRS	1.03	0.95	1.11	1.01	0.97	1.05	1.00	0.97	1.03	1.00	0.92	1.09
MDD PRS	1.09	1.01	1.19	1.04	0.99	1.09	1.04	1.00	1.08	1.04	0.95	1.14

*Note*: aOR, adjusted odds ratio; BPD, bipolar disorder; LCL, lower confidence limit; MDD, major depressive disorder; SCZ, schizophrenia; SD, standard deviation; UCL, upper confidence limit. The models were adjusted for sex, age, batch version, and 20 population stratification dimensions.

The mediation analyses of role of blood pressure and metabolic traits in the association of MDD PRS with myocardial infarction and PAD are presented in Supplementary Table 11. The results did not support the potential mediation role of blood pressure and metabolic traits. The low-density lipoprotein categories-stratified results for the MDD PRS association are presented in Supplementary Table 12. The results did not support the potential modifier role of low-density lipoprotein.

## Discussion

In this study, we used multiple approaches, including analyses of familial coaggregation and individual-level PRS, to explore the underlying mechanisms of the comorbidity between mental and cardiovascular disorders. Substantial studies confirm that mental and cardiovascular diseases each individually aggregate in families (Chou et al., 2017; Fischer et al., 2007; Parikh et al., 2007), but evidence for their familial coaggregation is lacking, except for a recent study based on Scandinavian populations (Meijsen et al., 2024). Here we provide the first comprehensive analysis from Asia of associations between three major mental disorders and four major cardiovascular diseases both within individuals and families using nationwide representative data. Our findings indicate familial coaggregation of these diseases, suggesting a shared familial risk. The PRS analysis revealed that genetic liability to MDD was positively associated with risk of cardiovascular diseases, particularly myocardial infarction and PAD, suggesting shared genetic loading between these diseases. No association was observed with genetic liability to schizophrenia or bipolar disorder.

Our analyses revealed co-occurrence of mental and cardiovascular diseases within families, even after adjusting for individuals’ mental disorders. Although familial coaggregation implies shared familial genetic, environmental, or both factors, our findings provide evidence of shared genetic risks of MDD–PAD comorbidity. First, the association in full siblings was similar to that observed in either parent (all sharing approximately 50% of their genes). Second, the familial association was stronger when both parents were affected with MDD than when either parent was affected. Third, the association was stronger in families with two affected siblings than in those with only one. Similar patterns were observed for schizophrenia–stroke and schizophrenia–PAD comorbidity, although the ORs were not statistically significant in certain subgroups probably due to a small number of cases.

Evidence indicates that environmental factors contributed more than the genetic factors underlying the comorbidity between mental and cardiometabolic disorders, and the relative contribution differs by disease pairs (Meijsen et al., 2024). Specifically, genetic components accounted for approximately 50% of comorbid manifestations of schizophrenia or affected disorders (mainly MDD) with cardiometabolic disorders (Meijsen et al., 2024). Both mental and cardiovascular diseases are complex diseases and likely share nongenetic factors, including behavior and external environmental influences within families. For example, lifestyle habits such as diet patterns, physical activity, and tobacco smoking, which are well-known cardiovascular risk factors that can also be passed intergenerationally (Jensen et al., 2021; Schwandt, Haas, & Liepold, 2010), are emerging as modifiable factors for prevention and treatment of mental illness (Firth et al., 2020). Family-based lifestyle interventions might be beneficial in reducing the risk of both mental and cardiovascular diseases.

Previous studies on PRS for mental disorders and cardiovascular diseases have primarily been conducted in European populations. The Dutch Lifelines cohort study reported a positive association between depression PRS and risk of atherosclerosis, whereas no such evidence was observed for schizophrenia or bipolar disorder (Veeneman et al., 2024). In line with these findings, our study showed that MDD PRS was positively associated with the risk of myocardial infarction and PAD, both atherosclerotic conditions. Other studies also support the genetic role in the association between depression and cardiovascular diseases, consistent with our observations (Bergstedt et al., 2024; Lu et al., 2021; Torgersen et al., 2022; Zhang, Cao, & Baranova, 2021). Generally, genome-wide genetic correlations between MDD and cardiovascular diseases, mainly coronary heart disease, stroke, PAD and heart failure, appeared to be moderate (Bergstedt et al., 2024; Lu et al., 2021; Torgersen et al., 2022; Zhang et al., 2021), and most risk variants for cardiovascular diseases were shared with MDD (Bergstedt et al., 2024; Zhang et al., 2021). Furthermore, Mendelian randomization analysis indicated causal effects of genetic liability to MDD on risk of cardiovascular diseases but found limited evidence on causal effects of cardiovascular diseases on MDD (Bergstedt et al., 2024; Lu et al., 2021).

Strengths of this study are the use of large-scale universal health insurance and individual genotyping data, and the latest GWAS discovery sample and advanced Bayesian methods to optimize PRS prediction and polygenic association. Moreover, disease information was ascertained using medical record diagnosis, which are likely more accurate than retrospectively self-reported data (Wu et al., 2024).

Our study has also limitations. First, we used NHI claims data from 1998 to 2020 to follow-up on study subjects and the diseases ascertainment. Therefore, only individuals who were alive during the study period were included. Disease diagnoses may have been missed if individuals did not seek medical care during this time frame. Second, body mass index, lifestyle behaviors, and psychotropic medication use were not considered in our familial coaggregation analysis. Their roles in the comorbidities and familial coaggregation between mental and cardiovascular diseases require further investigation. Our measures of potential mediators in the PRS association were limited by the cross-sectional nature. Longitudinal measures for blood pressure and metabolic traits before and after cardiovascular diseases onset were lacking. Although we attempted to explore the possible mediating effect of these modifiable covariates on the PRS association with cardiovascular outcomes, the causative pathway could not be clearly distinguished. Third, although the total sample size is large (*n* = 4.5 million in the overall cohort, 3.3 million in the full-sibling analysis, and around 92,000 to 106,000 in the PRS analysis), the statistical power may be insufficient for analyzing mental illnesses with low prevalence such as schizophrenia or bipolar disorder, where the number of cardiovascular events was small in some subgroups. Fourth, utilizing cross-ancestry GWAS results to generate PRS could reduce predictive performance, as genetic architecture varies across different populations (Martin et al., 2017). The discovery sample in this study was predominantly of European descent. To improve the polygenic prediction in Asian samples, we employed the PRS-CS/PRS-CSx approach to integrate GWAS summary statistics from various discovery samples. Moreover, PRS only considered common variants, thereby capturing only a limited portion of the heritability as estimated by family studies. Fifth, our data included only people of Taiwanese ancestry, which limits the generalizability of the findings to other populations. Sixth, conclusions about causality cannot be drawn, as our analysis was observational. However, our main aim was to assess familial coaggregation of mental and cardiovascular diseases. Further studies may benefit from applying causal analytic frameworks, such as target trial emulation, to strengthen causal inference in this association (Hernán & Robins, 2020).

## Conclusions

This nationwide population-based cohort study of over 4.5 million people revealed familial coaggregation of severe mental illness with major cardiovascular diseases, supporting the evidence for familial risk due to shared factors. Our PRS analysis helped clarify the shared polygenic risk specifically between MDD and myocardial infarction and PAD. These findings suggest the potential benefit of family-based integrated screening and preventive strategies that address both mental and cardiovascular health.

## Supporting information

10.1017/S0033291725101724.sm001 Chen et al. supplementary material Chen et al. supplementary material

## Data Availability

The NHIRD used in this study is held by the Taiwan Ministry of Health and Welfare. The genotyping data is available from the Taiwan Biobank. The authors are not allowed to distribute the data according to the ethical approval for this study.

## Supplementary material

The supplementary material for this article can be found at http://doi.org/10.1017/S0033291725101724.

## Table 1. Long description

Starting from the top, columns are labeled Total cohort and Full-sibling, each with subcolumns for n and percent. The total cohort has N equals 4,513,509 and the full-sibling cohort has N equals 3,330,181. The first variable is Sex, with Female comprising 2,125,041 or 47.08 percent of the total cohort and 1,608,636 or 48.30 percent of the full-sibling cohort. Male comprises 2,388,468 or 52.92 percent of the total cohort and 1,721,545 or 51.70 percent of the full-sibling cohort. Birth Cohort is next, with 1970 to 1979: 732,669 or 16.23 percent total, 391,344 or 11.75 percent full-sibling; 1980 to 1989: 1,716,252 or 38.02 percent total, 1,378,775 or 41.40 percent full-sibling; 1990 to 1999: 2,064,588 or 45.74 percent total, 1,560,062 or 46.85 percent full-sibling. Income level in New Taiwan Dollar is divided into less than 26,400: 903,679 or 20.05 percent total, 644,710 or 19.38 percent full-sibling; 26,400 to 45,800: 1,838,065 or 40.78 percent total, 1,370,104 or 41.19 percent full-sibling; greater than 45,800: 1,765,498 or 39.17 percent total, 1,311,104 or 39.42 percent full-sibling. Urbanization is categorized as Urban: 2,462,452 or 54.63 percent total, 1,776,746 or 53.41 percent full-sibling; Sub-urban: 1,677,108 or 37.21 percent total, 1,265,802 or 38.05 percent full-sibling; Rural: 368,078 or 8.17 percent total, 284,308 or 8.55 percent full-sibling. For mental disorders, Schizophrenia: 38,474 or 0.85 percent total, 41,186 or 1.24 percent full-sibling; Bipolar disorder: 49,351 or 1.09 percent total, 52,451 or 1.58 percent full-sibling; Major depressive disorder: 272,903 or 6.05 percent total, 279,726 or 8.40 percent full-sibling. NTD is defined as new Taiwan dollar.

Navigate back to Table 1..

## Table 2. Long description

The table has three main mental disorder categories: S C Z, B P D, and M D D. For each, rows are split by ‘No’ and ‘Yes’ for disorder presence. Columns are grouped by disease: myocardial infarction, stroke, peripheral arterial disease, and heart failure. For each disease, the columns are number of cases (n), percent, adjusted odds ratio (a O R), lower confidence limit (L C L), and upper confidence limit (U C L). For S C Z, those with the disorder have higher a O Rs for stroke (1.87), peripheral arterial disease (2.04), and heart failure (1.75) compared to those without (all 1.00), but not for myocardial infarction (0.90). For B P D, a O Rs are elevated for all diseases: myocardial infarction (1.36), stroke (2.38), peripheral arterial disease (2.39), and heart failure (1.72). For M D D, a O Rs are also higher: myocardial infarction (1.28), stroke (2.17), peripheral arterial disease (2.22), and heart failure (1.56). Confidence intervals are provided for each a O R. The reference group for all is those without the disorder (a O R 1.00). Sample sizes are largest for M D D, followed by B P D, then S C Z.

Navigate back to Table 2..

## Figure 1. Long description

Top left panel shows myocardial infarction. X-axis is odds ratio from 0 to 2.5. Groups are S C Z, B P D, and M D D, each split by paternal and maternal mental disorder status: P minus M minus, P plus M minus, P minus M plus, P plus M plus. For S C Z, Model 1 odds ratios range from 0.90 to 0.94, Model 2 from 0.90 to 0.95. For B P D, Model 1 ranges from 0.73 to 0.93, Model 2 from 0.73 to 0.90. For M D D, Model 1 ranges from 0.97 to 1.09, Model 2 from 0.89 to 0.99. Top right panel shows stroke with the same groupings. For S C Z, Model 1 odds ratios range from 1.09 to 2.20, Model 2 from 1.06 to 1.97. For B P D, Model 1 ranges from 1.11 to 1.18, Model 2 from 1.07 to 1.07. For M D D, Model 1 ranges from 1.09 to 1.13, Model 2 from 1.06 to 1.09. Bottom left panel shows peripheral arterial disease. For S C Z, Model 1 odds ratios range from 1.19 to 2.24, Model 2 from 1.16 to 1.96. For B P D, Model 1 ranges from 0.99 to 1.45, Model 2 from 0.96 to 1.32. For M D D, Model 1 ranges from 1.10 to 1.33, Model 2 from 1.05 to 1.27. Bottom right panel shows heart failure. For S C Z, Model 1 odds ratios range from 1.05 to 1.38, Model 2 from 1.02 to 1.26. For B P D, Model 1 ranges from 0.89 to 1.33, Model 2 from 0.87 to 1.32. For M D D, Model 1 ranges from 1.00 to 1.07, Model 2 from 0.99 to 1.07. Across all panels, the highest odds ratios are observed for S C Z with both parents affected, especially for stroke and peripheral arterial disease.

Navigate back to Figure 1..

## Figure 2. Long description

Top left panel A shows myocardial infarction. For siblings with schizophrenia, odds ratios are near 1 for both models. For bipolar disorder, odds ratios are slightly above 1, with higher values for two or more siblings. For major depressive disorder, odds ratios are above 1, especially for two or more siblings, reaching 1.75 in Model 1 and 1.69 in Model 2. Top right panel B shows stroke. Odds ratios for schizophrenia and bipolar disorder are close to 1. For major depressive disorder, odds ratios are slightly above 1, but confidence intervals cross 1. Bottom left panel C shows peripheral arterial disease. Odds ratios for schizophrenia are above 1, especially for two or more siblings. For bipolar disorder, odds ratios are near 1. For major depressive disorder, odds ratios are above 1, with two or more siblings showing higher values. Bottom right panel D shows heart failure. Odds ratios for schizophrenia and bipolar disorder are near 1. For major depressive disorder, odds ratios are above 1, with two or more siblings showing the highest values. All panels display odds ratios with 95 percent confidence intervals for Model 1 and Model 2, with a reference line at 1. Labels include a O R, 95 percent C I, S C Z, B P D, M D D, and odds ratio scale from 0.5 to 2.5.

Navigate back to Figure 2..

## Table 3. Long description

From left to right, the first column lists polygenic risk scores: S C Z P R S, B P D P R S, and M D D P R S. For each, four cardiovascular outcomes are shown in grouped columns: myocardial infarction, stroke, peripheral arterial disease, and heart failure. Each outcome has three subcolumns: a O R (adjusted odds ratio), L C L (lower confidence limit), and U C L (upper confidence limit). For S C Z P R S, myocardial infarction a O R is 1.00, L C L 0.93, U C L 1.08; stroke a O R 1.01, L C L 0.96, U C L 1.05; peripheral arterial disease a O R 0.99, L C L 0.96, U C L 1.02; heart failure a O R 0.94, L C L 0.87, U C L 1.02. For B P D P R S, myocardial infarction a O R is 1.03, L C L 0.95, U C L 1.11; stroke a O R 1.01, L C L 0.97, U C L 1.05; peripheral arterial disease a O R 1.00, L C L 0.97, U C L 1.03; heart failure a O R 1.00, L C L 0.92, U C L 1.09. For M D D P R S, myocardial infarction a O R is 1.09, L C L 1.01, U C L 1.19; stroke a O R 1.04, L C L 0.99, U C L 1.09; peripheral arterial disease a O R 1.04, L C L 1.00, U C L 1.08; heart failure a O R 1.04, L C L 0.95, U C L 1.14. The models are adjusted for sex, age, batch version, and 20 population stratification dimensions.

Navigate back to Table 3..
