# Supplementary material for: Familial coaggregation and shared genetic loading of mental disorders and cardiovascular diseases
Source: Psychol Med. 2026 May 28;56:e164. doi: 10.1017/S0033291725101724 (PMC13234514; doi:10.1017/S0033291725101724)
Supplement: Chen et al. supplementary material [file S0033291725101724sup001.docx]

Supplementary table 1. Disease diagnosis codes

| Diseases | ICD 9 | ICD 10 | Criterion |
| --- | --- | --- | --- |
| Schizophrenia | 295.x | F20, F25 | 2 outpatient visits and/or 1 inpatient admission |
| Bipolar disorder | 296.0-296.1, 296.4-296.8 | F30, F31, F34.0, | 2 outpatient visits and/or 1 inpatient admission |
| Major depressive disorder | 296.2x, 296.3x, 300.4, 311 | F32, F33, F34.1 | 2 outpatient visits and/or 1 inpatient admission |
| Myocardial infarction | 410 | I21-I22 | Any inpatient admission |
| Stroke | 430-438 | I60-I69 | Any inpatient admission |
| Peripheral arterial disease | 440.0, 440.2, 440.3, 440.8, 440.9, 443.9, 444.0, 444.2, 444.8, 447.8, 447.9 | I70.0, I70.2, I70.3, I70.4, I70.5, I70.6, I70.7, I70.8, I70.9, I73.9, I74.01, I74.09, I74.2, I74.3, I74.4, I74.5, I74.8, I75.01, I75.02, I75.89, I77.3, I77.89, I77.9 | 2 outpatient visits and/or 1 inpatient admission |
| Heart failure | 428 | I50 | Any inpatient admission |

| Supplementary table 2. Odds ratios of myocardial infarction in association with parental history of mental disorders (sample size=4,513,509). | | | | | | | | | | | | |
| --- | --- | --- | --- | --- | --- | --- | --- | --- | --- | --- | --- | --- |
| Paternal (P)/maternal (M) mental disorders | N | n | % | Model 1 | | | |  | Model 2 | | | |
|  |  |  |  | aOR | LCL | UCL | P value |  | aOR | LCL | UCL | P value |
| SCZ |  |  |  |  |  |  |  |  |  |  |  |  |
| P-M- | 4441370 | 4388 | 0.10 | 1.00 | - | - |  |  | 1.00 | - | - |  |
| P+M- | 26551 | 23 | 0.09 | 0.90 | 0.60 | 1.36 | 0.6176 |  | 0.90 | 0.60 | 1.36 | 0.6297 |
| P-M+ | 44590 | 46 |  | 0.94 | 0.70 | 1.26 | 0.6604 |  | 0.94 | 0.70 | 1.27 | 0.6890 |
| P+M+ | 998 |  |  | 0.94 | 0.13 | 6.72 | 0.9503 |  | 0.95 | 0.13 | 6.84 | 0.9634 |
| BPD |  |  |  |  |  |  |  |  |  |  |  |  |
| P-M- | 4384435 | 4335 | 0.10 | 1.00 | - | - |  |  | 1.00 | - | - |  |
| P+M- | 48511 | 37 | 0.08 | 0.73 | 0.53 | 1.02 | 0.0617 |  | 0.73 | 0.53 | 1.01 | 0.0547 |
| P-M+ | 76916 | 81 | 0.11 | 1.02 | 0.82 | 1.28 | 0.8318 |  | 1.01 | 0.81 | 1.27 | 0.9125 |
| P+M+ | 3647 | 4 | 0.11 | 0.93 | 0.35 | 2.49 | 0.8868 |  | 0.90 | 0.34 | 2.41 | 0.8413 |
| MDD |  |  |  |  |  |  |  |  |  |  |  |  |
| P-M- | 3570033 | 3380 | 0.09 | 1.00 | - | - |  |  | 1.00 | - | - |  |
| P+M- | 300959 | 364 | 0.12 | 1.08 | 0.97 | 1.21 | 0.1623 |  | 1.07 | 0.96 | 1.19 | 0.2209 |
| P-M+ | 541648 | 594 | 0.11 | 0.97 | 0.89 | 1.06 | 0.5360 |  | 0.96 | 0.88 | 1.05 | 0.3769 |
| P+M+ | 100869 | 119 | 0.12 | 0.91 | 0.76 | 1.09 | 0.3080 |  | 0.89 | 0.74 | 1.06 | 0.1932 |
| Abbreviations: aOR, adjusted odds ratio; BPD, bipolar disorder; LCL, lower confidence limit; MDD, major depressive disorder; SCZ, schizophrenia; UCL, upper confidence limit.  Model 1 was adjusted for sex, birth cohort, age, income level, urbanization level, father’s age, and mother’s age. | | | | | | | | | | | | |
| Model 2 was further adjusted for the individual’s corresponding psychiatric diagnosis. | | | | | | | | | | | | |

| Supplementary table 3. Odds ratios of stroke in association with parental history of mental disorders (sample size=4,513,509). | | | | | | | | | | | | |  |
| --- | --- | --- | --- | --- | --- | --- | --- | --- | --- | --- | --- | --- | --- |
| Paternal (P)/maternal (M) mental disorders | N | n | % | Model 1 | | | |  | Model 2 | | | | |
|  |  |  |  | aOR | LCL | UCL | P value |  | aOR | LCL | UCL | P value | |
| SCZ |  |  |  |  |  |  |  |  |  |  |  |  | |
| P-M- | 4441370 | 16989 | 0.38 | 1.00 | - | - |  |  | 1.00 | - | - |  | |
| P+M- | 26551 | 112 | 0.42 | 1.09 | 0.90 | 1.31 | 0.3697 |  | 1.06 | 0.88 | 1.28 | 0.5368 | |
| P-M+ | 44590 | 212 | 0.48 | 1.13 | 0.99 | 1.30 | 0.0676 |  | 1.08 | 0.95 | 1.24 | 0.2406 | |
| P+M+ | 998 | 9 | 0.90 | 2.20 | 1.14 | 4.26 | 0.0188 |  | 1.97 | 1.01 | 3.82 | 0.0453 | |
| BPD |  |  |  |  |  |  |  |  |  |  |  |  | |
| P-M- | 4384435 | 16732 | 0.38 | 1.00 | - | - |  |  | 1.00 | - | - |  | |
| P+M- | 48511 | 211 | 0.43 | 1.11 | 0.97 | 1.27 | 0.1449 |  | 1.07 | 0.94 | 1.23 | 0.3074 | |
| P-M+ | 76916 | 361 | 0.47 | 1.20 | 1.08 | 1.34 | 0.0006 |  | 1.16 | 1.04 | 1.28 | 0.0067 | |
| P+M+ | 3647 | 18 | 0.49 | 1.18 | 0.74 | 1.87 | 0.4899 |  | 1.07 | 0.67 | 1.70 | 0.7725 | |
| MDD |  |  |  |  |  |  |  |  |  |  |  |  | |
| P-M- | 3570033 | 13071 | 0.37 | 1.00 | - | - |  |  | 1.00 | - | - |  | |
| P+M- | 300959 | 1311 | 0.44 | 1.09 | 1.03 | 1.16 | 0.0027 |  | 1.06 | 1.00 | 1.12 | 0.0570 | |
| P-M+ | 541648 | 2448 | 0.45 | 1.13 | 1.09 | 1.18 | <.0001 |  | 1.08 | 1.04 | 1.13 | 0.0003 | |
| P+M+ | 100869 | 492 | 0.49 | 1.13 | 1.03 | 1.24 | 0.0080 |  | 1.03 | 0.94 | 1.13 | 0.5379 | |
| Abbreviations: aOR, adjusted odds ratio; BPD, bipolar disorder; LCL, lower confidence limit; MDD, major depressive disorder; SCZ, schizophrenia; UCL, upper confidence limit.  Model 1 was adjusted for sex, birth cohort, age, income level, urbanization level, father’s age, and mother’s age. | | | | | | | | | | | | |  |
| Model 2 was further adjusted for the individual’s corresponding psychiatric diagnosis. | | | | | | | | | | | | |  |

| Supplementary table 4. Odds ratios of peripheral arterial disease in association with parental history of mental disorders (sample size=4,513,509). | | | | | | | | | | | | |
| --- | --- | --- | --- | --- | --- | --- | --- | --- | --- | --- | --- | --- |
| Paternal (P)/maternal (M) mental disorders | N | n | % | Model 1 | | | |  | Model 2 | | | |
|  |  |  |  | aOR | LCL | UCL | P value |  | aOR | LCL | UCL | P value |
| SCZ |  |  |  |  |  |  |  |  |  |  |  |  |
| P-M- | 4441370 | 17731 | 0.40 | 1.00 | - | - |  |  | 1.00 | - | - |  |
| P+M- | 26551 | 124 | 0.47 | 1.19 | 1.00 | 1.42 | 0.0554 |  | 1.16 | 0.97 | 1.38 | 0.1093 |
| P-M+ | 44590 | 257 | 0.58 | 1.37 | 1.21 | 1.55 | <.0001 |  | 1.30 | 1.15 | 1.48 | <.0001 |
| P+M+ | 998 | 9 | 0.90 | 2.24 | 1.08 | 4.65 | 0.0298 |  | 1.96 | 0.95 | 4.07 | 0.0698 |
| BPD |  |  |  |  |  |  |  |  |  |  |  |  |
| P-M- | 4384435 | 17463 | 0.40 | 1.00 | - | - |  |  | 1.00 | - | - |  |
| P+M- | 48511 | 197 | 0.41 | 0.99 | 0.86 | 1.14 | 0.8440 |  | 0.96 | 0.83 | 1.10 | 0.5476 |
| P-M+ | 76916 | 438 | 0.57 | 1.40 | 1.27 | 1.55 | <.0001 |  | 1.35 | 1.22 | 1.49 | <.0001 |
| P+M+ | 3647 | 23 | 0.63 | 1.45 | 0.94 | 2.22 | 0.0911 |  | 1.32 | 0.86 | 2.03 | 0.2059 |
| MDD |  |  |  |  |  |  |  |  |  |  |  |  |
| P-M- | 3570033 | 13423 | 0.38 | 1.00 | - | - |  |  | 1.00 | - | - |  |
| P+M- | 300959 | 1372 | 0.46 | 1.10 | 1.04 | 1.16 | 0.0009 |  | 1.06 | 1.01 | 1.13 | 0.0302 |
| P-M+ | 541648 | 2719 | 0.50 | 1.21 | 1.16 | 1.26 | <.0001 |  | 1.15 | 1.10 | 1.20 | <.0001 |
| P+M+ | 100869 | 607 | 0.60 | 1.33 | 1.22 | 1.44 | <.0001 |  | 1.20 | 1.11 | 1.31 | <.0001 |
| Abbreviations: aOR, adjusted odds ratio; BPD, bipolar disorder; LCL, lower confidence limit; MDD, major depressive disorder; SCZ, schizophrenia; UCL, upper confidence limit.  Model 1 was adjusted for sex, birth cohort, age, income level, urbanization level, father’s age, and mother’s age. | | | | | | | | | | | | |
| Model 2 was further adjusted for the individual’s corresponding psychiatric diagnosis. | | | | | | | | | | | | |

| Supplementary table 5. Odds ratios of heart failure in association with parental history of mental disorders (sample size=4,513,509). | | | | | | | | | | | | |
| --- | --- | --- | --- | --- | --- | --- | --- | --- | --- | --- | --- | --- |
| Paternal (P)/maternal (M) mental disorders | N | n | % | Model 1 | | | |  | Model 2 | | | |
|  |  |  |  | aOR | LCL | UCL | P value |  | aOR | LCL | UCL | P value |
| SCZ |  |  |  |  |  |  |  |  |  |  |  |  |
| P-M- | 4441370 | 8499 | 0.19 | 1.00 | - | - |  |  | 1.00 | - | - |  |
| P+M- | 26551 | 68 | 0.26 | 1.27 | 1.00 | 1.61 | 0.0524 |  | 1.24 | 0.98 | 1.58 | 0.0793 |
| P-M+ | 44590 | 99 | 0.22 | 1.05 | 0.86 | 1.28 | 0.6612 |  | 1.01 | 0.82 | 1.23 | 0.9563 |
| P+M+ | 998 | 3 | 0.30 | 1.38 | 0.44 | 4.28 | 0.5772 |  | 1.26 | 0.41 | 3.92 | 0.6887 |
| BPD |  |  |  |  |  |  |  |  |  |  |  |  |
| P-M- | 4384435 | 8396 | 0.19 | 1.00 | - | - |  |  | 1.00 | - | - |  |
| P+M- | 48511 | 84 | 0.17 | 0.89 | 0.72 | 1.10 | 0.2734 |  | 0.87 | 0.70 | 1.08 | 0.2144 |
| P-M+ | 76916 | 179 | 0.23 | 1.19 | 1.02 | 1.38 | 0.0245 |  | 1.16 | 1.00 | 1.35 | 0.0478 |
| P+M+ | 3647 | 10 | 0.27 | 1.33 | 0.72 | 2.48 | 0.3629 |  | 1.27 | 0.68 | 2.36 | 0.4514 |
| MDD |  |  |  |  |  |  |  |  |  |  |  |  |
| P-M- | 3570033 | 6699 | 0.19 | 1.00 | - | - |  |  | 1.00 | - | - |  |
| P+M- | 300959 | 649 | 0.22 | 1.09 | 1.00 | 1.18 | 0.0401 |  | 1.07 | 0.99 | 1.16 | 0.0938 |
| P-M+ | 541648 | 1092 | 0.20 | 1.02 | 0.96 | 1.09 | 0.5410 |  | 1.00 | 0.93 | 1.06 | 0.9420 |
| P+M+ | 100869 | 229 | 0.23 | 1.07 | 0.94 | 1.23 | 0.3138 |  | 1.02 | 0.89 | 1.17 | 0.7573 |
| Abbreviations: aOR, adjusted odds ratio; BPD, bipolar disorder; LCL, lower confidence limit; MDD, major depressive disorder; SCZ, schizophrenia; UCL, upper confidence limit.  Model 1 was adjusted for sex, birth cohort, age, income level, urbanization level, father’s age, and mother’s age. | | | | | | | | | | | | |
| Model 2 was further adjusted for the individual’s corresponding psychiatric diagnosis. | | | | | | | | | | | | |

| Supplementary table 6. Odds ratios of myocardial infarction in association with full siblings’ history of mental disorders (sample size=3,330,181). | | | | | | | | | | | | | |
| --- | --- | --- | --- | --- | --- | --- | --- | --- | --- | --- | --- | --- | --- |
| Mental disorders of full sibling's | | N | n | % | Model 1 | | | |  | Model 2 | | | |
|  |  |  |  |  | aOR | LCL | UCL | P value |  | aOR | LCL | UCL | P value |
| SCZ | No | 3288995 | 2446 | 0.07 | 1.00 | - | - |  |  | 1.00 | - | - |  |
|  | Yes | 41186 | 41 | 0.10 | 0.99 | 0.73 | 1.35 | 0.9602 |  | 1.00 | 0.73 | 1.36 | 0.9918 |
| BPD | No | 3277730 | 2438 | 0.07 | 1.00 | - | - |  |  | 1.00 | - | - |  |
|  | Yes | 52451 | 49 | 0.09 | 1.10 | 0.83 | 1.46 | 0.5032 |  | 1.09 | 0.82 | 1.45 | 0.5528 |
| MDD | No | 3050455 | 2211 | 0.07 | 1.00 | - | - |  |  | 1.00 | - | - |  |
|  | Yes | 279726 | 276 | 0.10 | 1.14 | 1.00 | 1.29 | 0.0435 |  | 1.12 | 0.99 | 1.27 | 0.0746 |
| Abbreviations: aOR, adjusted odds ratio; BPD, bipolar disorder; LCL, lower confidence limit; MDD, major depressive disorder; SCZ, schizophrenia; UCL, upper confidence limit.  Model 1 was adjusted for sex, birth cohort, age, income level, urbanization level, sibling’s age, and sibling size. | | | | | | | | | | | | | |
| Model 2 was further adjusted for the individual’s corresponding psychiatric diagnosis. | | | | | | | | | | | | | |

| Supplementary table 7. Odds ratios of stroke in association with full siblings’ history of mental disorders (sample size=3,330,181). | | | | | | | | | | | | | |
| --- | --- | --- | --- | --- | --- | --- | --- | --- | --- | --- | --- | --- | --- |
| Mental disorders of full sibling's | | N | n | % | Model 1 | | | |  | Model 2 | | | |
|  |  |  |  |  | aOR | LCL | UCL | P value |  | aOR | LCL | UCL | P value |
| SCZ | No | 3288995 | 11256 | 0.34 | 1.00 | - | - |  |  | 1.00 | - | - |  |
|  | Yes | 41186 | 194 | 0.47 | 1.10 | 0.95 | 1.27 | 0.1980 |  | 1.05 | 0.91 | 1.21 | 0.5147 |
| BPD | No | 3277730 | 11234 | 0.34 | 1.00 | - | - |  |  | 1.00 | - | - |  |
|  | Yes | 52451 | 216 | 0.41 | 1.08 | 0.94 | 1.23 | 0.3016 |  | 1.03 | 0.90 | 1.19 | 0.6345 |
| MDD | No | 3050455 | 10284 | 0.34 | 1.00 | - | - |  |  | 1.00 | - | - |  |
|  | Yes | 279726 | 1166 | 0.42 | 1.07 | 1.01 | 1.14 | 0.0222 |  | 1.01 | 0.95 | 1.08 | 0.6577 |
| Abbreviations: aOR, adjusted odds ratio; BPD, bipolar disorder; LCL, lower confidence limit; MDD, major depressive disorder; SCZ, schizophrenia; UCL, upper confidence limit.  Model 1 was adjusted for sex, birth cohort, age, income level, urbanization level, sibling’s age, and sibling size. | | | | | | | | | | | | | |
| Model 2 was further adjusted for the individual’s corresponding psychiatric diagnosis. | | | | | | | | | | | | | |

| Supplementary table 8. Odds ratios of peripheral arterial disease in association with full siblings’ history of mental disorders (sample size=3,330,181). | | | | | | | | | | | | | |
| --- | --- | --- | --- | --- | --- | --- | --- | --- | --- | --- | --- | --- | --- |
| Mental disorders of full sibling's | | N | n | % | Model 1 | | | |  | Model 2 | | | |
|  |  |  |  |  | aOR | LCL | UCL | P value |  | aOR | LCL | UCL | P value |
| SCZ | No | 3288995 | 11884 | 0.36 | 1.00 | - | - |  |  | 1.00 | - | - |  |
|  | Yes | 41186 | 220 | 0.53 | 1.18 | 1.03 | 1.36 | 0.0176 |  | 1.16 | 1.01 | 1.33 | 0.0365 |
| BPD | No | 3277730 | 11866 | 0.36 | 1.00 | - | - |  |  | 1.00 | - | - |  |
|  | Yes | 52451 | 238 | 0.45 | 1.08 | 0.95 | 1.24 | 0.2495 |  | 1.08 | 0.95 | 1.23 | 0.2468 |
| MDD | No | 3050455 | 10788 | 0.35 | 1.00 | - | - |  |  | 1.00 | - | - |  |
|  | Yes | 279726 | 1316 | 0.47 | 1.14 | 1.07 | 1.21 | <.0001 |  | 1.10 | 1.03 | 1.16 | 0.0024 |
| Abbreviations: aOR, adjusted odds ratio; BPD, bipolar disorder; LCL, lower confidence limit; MDD, major depressive disorder; SCZ, schizophrenia; UCL, upper confidence limit.  Model 1 was adjusted for sex, birth cohort, age, income level, urbanization level, sibling’s age, and sibling size. | | | | | | | | | | | | | |
| Model 2 was further adjusted for the individual’s corresponding psychiatric diagnosis. | | | | | | | | | | | | | |

| Supplementary table 9. Odds ratios of heart failure in association with full siblings’ history of mental disorders (sample size=3,330,181). | | | | | | | | | | | | | |
| --- | --- | --- | --- | --- | --- | --- | --- | --- | --- | --- | --- | --- | --- |
| Mental disorders of full sibling's | | N | n | % | Model 1 | | | |  | Model 2 | | | |
|  |  |  |  |  | aOR | LCL | UCL | P value |  | aOR | LCL | UCL | P value |
| SCZ | No | 3288995 | 5608 | 0.17 | 1.00 | - | - |  |  | 1.00 | - | - |  |
|  | Yes | 41186 | 103 | 0.25 | 1.19 | 0.98 | 1.44 | 0.0869 |  | 1.15 | 0.95 | 1.40 | 0.1506 |
| BPD | No | 3277730 | 5604 | 0.17 | 1.00 | - | - |  |  | 1.00 | - | - |  |
|  | Yes | 52451 | 107 | 0.20 | 1.08 | 0.89 | 1.31 | 0.4313 |  | 1.06 | 0.88 | 1.29 | 0.5380 |
| MDD | No | 3050455 | 5115 | 0.17 | 1.00 | - | - |  |  | 1.00 | - | - |  |
|  | Yes | 279726 | 596 | 0.21 | 1.12 | 1.03 | 1.22 | 0.0096 |  | 1.09 | 1.00 | 1.19 | 0.0434 |
| Abbreviations: aOR, adjusted odds ratio; BPD, bipolar disorder; LCL, lower confidence limit; MDD, major depressive disorder; SCZ, schizophrenia; UCL, upper confidence limit.  Model 1 was adjusted for sex, birth cohort, age, income level, urbanization level, sibling’s age, and sibling size. | | | | | | | | | | | | | |
| Model 2 was further adjusted for the individual’s corresponding psychiatric diagnosis. | | | | | | | | | | | | | |

| Supplementary table 10. Distribution of the demographics, mental disorders, and cardiovascular diseases in 106796 participants of the Taiwan Biobank. | |
| --- | --- |
| Variable | n (%) |
|  |  |
|  |  |
| Sex male | 38914 (36.44) |
| Age at enrollment, years |  |
| 30-39 | 23454 (21.96) |
| 40-49 | 27141 (25.41) |
| 50-59 | 32716 (30.63) |
| >=60 | 23485 (21.99) |
| Psychiatric disorders |  |
| Schizophrenia | 669 (0.63) |
| bipolar disorder | 1725 (1.62) |
| Major depressive disorder | 13968 (13.08) |
| Cardiovascular diseases |  |
| Myocardial infarction | 659 (0.62) |
| Stoke | 2176 (2.04) |
| Heart failure | 583 (0.55) |
| Peripheral arterial disease | 4148 (3.88) |
| Metabolic and cardiovascular traits |  |
| BMI, mean ± SD | 24.26 ± 3.80 |
| SBP, mean ± SD | 119 ± 17.79 |
| DBP, mean ± SD | 73 ± 11.01 |
| TG, mean ± SD | 116 ± 95.41 |
| TC, mean ± SD | 196 ± 37.30 |
| HDL, mean ± SD | 55 ± 13.44 |
| LDL, mean ± SD | 121 ± 31.76 |
| LDL<1.0 mmol/L | 164 (0.15) |
| LDL<1.4 mmol/L | 973 (0.91) |

Supplementary table 11. Mediation effects of metabolic and cardiovascular traits on the association of polygenic risk score for MDD with MI and PAD.

| Mediator | Outcome: MI | | | | | | | | |
| --- | --- | --- | --- | --- | --- | --- | --- | --- | --- |
|  | Direct effect | | | Indirect effect | | | Mediation proportion | | |
|  | Estimate | LCL | UCL | Estimate | LCL | UCL | Estimate | LCL | UCL |
| BMI | 1.067 | 0.996 | 1.139 | 1.001 | 0.999 | 1.003 | 1.345 | -2.160 | 4.850 |
| SBP | 1.067 | 0.990 | 1.145 | 0.999 | 0.998 | 1.001 | -1.214 | -3.788 | 1.360 |
| DBP | 1.073 | 0.997 | 1.148 | 1.000 | 0.999 | 1.001 | 0.087 | -1.230 | 1.404 |
| TG | 1.061 | 0.989 | 1.132 | 1.000 | 1.000 | 1.001 | 0.444 | -0.888 | 1.776 |
| HDL | 1.070 | 0.997 | 1.143 | 1.001 | 0.997 | 1.004 | 1.093 | -3.948 | 6.134 |
| LDL | 1.070 | 0.999 | 1.141 | 1.001 | 1.000 | 1.002 | 0.848 | -0.928 | 2.625 |
| TC | 1.069 | 0.998 | 1.140 | 1.001 | 1.000 | 1.002 | 1.335 | -0.883 | 3.553 |
| Mediator | Outcome: PAD | | | | | | | | |
|  | Direct effect | | | Indirect effect | | | Mediation proportion | | |
|  | Estimate | LCL | UCL | Estimate | LCL | UCL | Estimate | LCL | UCL |
| BMI | 1.036 | 1.007 | 1.066 | 1.000 | 0.999 | 1.002 | 1.212 | -1.859 | 4.282 |
| SBP | 1.038 | 1.007 | 1.068 | 1.000 | 1.000 | 1.000 | -0.519 | -1.562 | 0.524 |
| DBP | 1.037 | 1.007 | 1.067 | 1.000 | 1.000 | 1.000 | 0.039 | -0.547 | 0.624 |
| TG | 1.037 | 1.007 | 1.066 | 1.000 | 1.000 | 1.001 | 0.394 | -0.748 | 1.536 |
| HDL | 1.037 | 1.007 | 1.066 | 1.000 | 1.000 | 1.001 | 0.372 | -1.345 | 2.090 |
| LDL | 1.036 | 1.007 | 1.066 | 1.000 | 1.000 | 1.001 | 0.500 | -0.531 | 1.531 |
| TC | 1.040 | 1.010 | 1.070 | 1.000 | 1.000 | 1.001 | 0.674 | -0.385 | 1.732 |

Supplementary table 12. The estimated association of polygenic risk score for MDD with MI and PAD, stratified by LDL.

|  | Outcome: MI | | | | Outcome: PAD | | | |
| --- | --- | --- | --- | --- | --- | --- | --- | --- |
|  | Estimate | LCL | UCL | p-value | Estimate | LCL | UCL | p-value |
| LDL<1.4 mmol/L | 1.177 | 0.791 | 1.752 | 0.420 | 0.925 | 0.689 | 1.241 | 0.603 |
| LDL>=1.4 mmol/L | 1.072 | 1.001 | 1.147 | 0.045 | 1.037 | 1.008 | 1.068 | 0.013 |
| Z test |  |  |  | 0.672 |  |  |  | 0.429 |
